# Metabolic Fingerprinting to Assess the Impact of Salinity on Carotenoid Content in Developing Tomato Fruits

**DOI:** 10.3390/ijms17060821

**Published:** 2016-05-26

**Authors:** Lieven Van Meulebroek, Jochen Hanssens, Kathy Steppe, Lynn Vanhaecke

**Affiliations:** 1 Laboratory of Chemical Analysis, Department of Veterinary Public Health and Food Safety, Faculty of Veterinary Medicine, Ghent University, Salisburylaan 133, 9820 Merelbeke, Belgium; Lieven.Vanmeulenbroek@UGent.be; 2 Laboratory of Plant Ecology, Department of Applied Ecology and Plant Biology, Faculty of Bioscience Engineering, Ghent University, Coupure Links 653, 9000 Gent, Belgium; jochen.hanssens@Inagro.be (J.H.); Kathy.Steppe@UGent.be (K.S.)

**Keywords:** tomato (*Solanum lycopersicum* L.), metabolic fingerprinting, mass spectrometry, salinity, carotenoids, phytohormones

## Abstract

As the presence of health-promoting substances has become a significant aspect of tomato fruit appreciation, this study investigated nutrient solution salinity as a tool to enhance carotenoid accumulation in cherry tomato fruit (*Solanum lycopersicum* L. cv. Juanita). Hereby, a key objective was to uncover the underlying mechanisms of carotenoid metabolism, moving away from typical black box research strategies. To this end, a greenhouse experiment with five salinity treatments (ranging from 2.0 to 5.0 decisiemens (dS) m^−1^) was carried out and a metabolomic fingerprinting approach was applied to obtain valuable insights on the complicated interactions between salinity treatments, environmental conditions, and the plant’s genetic background. Hereby, several hundreds of metabolites were attributed a role in the plant’s salinity response (at the fruit level), whereby the overall impact turned out to be highly depending on the developmental stage. In addition, 46 of these metabolites embraced a dual significance as they were ascribed a prominent role in carotenoid metabolism as well. Based on the specific mediating actions of the retained metabolites, it could be determined that altered salinity had only marginal potential to enhance carotenoid accumulation in the concerned tomato fruit cultivar. This study invigorates the usefulness of metabolomics in modern agriculture, for instance in modeling tomato fruit quality. Moreover, the metabolome changes that were caused by the different salinity levels may enclose valuable information towards other salinity-related plant processes as well.

## 1. Introduction

Tomato (*Solanum lycopersicum* L.) is one of the most important horticultural crops worldwide and assigned a key role in human diet. Moreover, as a major component of daily meals in many countries, tomato is an important source of diverse health-beneficial constituents [1] and its consumption has been associated with a reduced incidence of various chronic degenerative diseases [2,3]. As such, the presence of health-promoting substances has become an important aspect for tomato fruit quality appreciation and of growing interest for consumers [4]. Improving nutritional quality has thus emerged as a challenge for growers who want to meet the ever-increasing demands of consumers in a highly competitive fresh market [5]. Therefore, a major part of current research focuses on the identification of agronomic means that may enhance the content of bioactive compounds in tomato fruit [6]. Particularly, the carotenoids lycopene and β-carotene are considered as target compounds because of their predominant contribution to overall tomato fruit nutritional quality [7].

In this context, management of the nutrient solution electrical conductivity (EC) has been intensively assessed as a strategy to enhance tomato fruit carotenoid content. It has indeed been postulated that an increased EC-level may be beneficial to crops because of an improved nutritional value [8,9]. Initially, improvements were exclusively attributed to a concentration effect of compounds in the tomato fruit, being the result of osmotic stress and related impaired water uptake by the fruits. However, physiological plant responses may be activated as well [10,11]. For example, in the study of Wu *et al.* (2008) [5], lycopene concentration levels were enhanced when tomato plants were grown hydroponically using a nutrient solution with an EC-level of 4.8 dS m^−1^ compared to standard EC-conditions of 2.4 dS m^−1^. Moreover, the increase in lycopene concentration (34% to 85%) for the four cultivars tested was significantly higher than the increase in total soluble solids (12% to 22%). As such, these findings suggest an improved nutritional quality because of specific plant stress responses, rather than being the result of reduced tomato fruit water content. However, although various studies have ascertained positive effects of this agronomic measure [8,12,13], conflicting or less conclusive data have been reported as well [14,15,16]. After all, the final carotenoid content strongly depends on the interactions between agronomic setting, environment, and genetic background [17]. Consequently, defining optimum conditions to maximize carotenoid biosynthesis or accumulation is difficult or may even be impossible [18]. In this regard, predictive and mechanistic fruit quality models are interesting since they may assist in setting conditions that are appropriate to reach high tomato fruit nutritional quality. However, relatively little progress has been made in modeling fruit quality because of the system’s complexity and the underlying mechanisms of metabolism, which are only partially understood [19]. A deepened physiological and biochemical knowledge on the metabolism of carotenoids in relation to their regulation and growth environment is thus essential in the pursuit of higher nutritional value. Phytohormones could be of special interest here as their regulating actions may enfold the missing link between growth environment and carotenoid metabolism. These biomolecules fulfill a crucial role in the regulation of the plant response to environmental stimuli [20] and are involved in the complex processes of fruit development and ripening, which are accompanied by qualitative and quantitative changes in the carotenoid profile [21].

In this study, nutrient solution salinity was assessed as a practice to promote tomato carotenoid content and thus improve nutritional quality. To this end, a metabolic fingerprinting strategy was employed to characterize the metabolome changes, provoked by the imposed salinity treatments, and underlying alterations in carotenoid metabolism. New insights on the regulatory mechanisms of carotenoid metabolism may lead to a more informed decision on the true potential of salinity, hereby moving away from the typical black box scenarios. In this context, phytohormones are of particular interest because of their short-term reactions to environmental changes, which could be fully exploited in optimizing and modeling tomato fruit quality. Therefore, metabolic fingerprinting was oriented to the polar metabolome fraction in order to cover the predominantly polar phytohormones the most. Fingerprints were acquired through full-scan high-resolution (hybrid quadrupole) Orbitrap mass spectrometry (MS). In this study, several hundreds (>300) of polar metabolites were assigned a potential role in the salinity response at the tomato fruit level. From this metabolome subset, various metabolites were linked to carotenoid metabolism, whereby it was concluded that the final effect of salinity was marginal for this tomato cultivar. Based on this metabolic approach, it could be assumed that salinity has only little potential to improve nutritional quality for this cultivar, even when environmental conditions would be different.

## 2. Results

### 2.1. Effectiveness of Imposed Salinity Treatments

**Figure 1:**
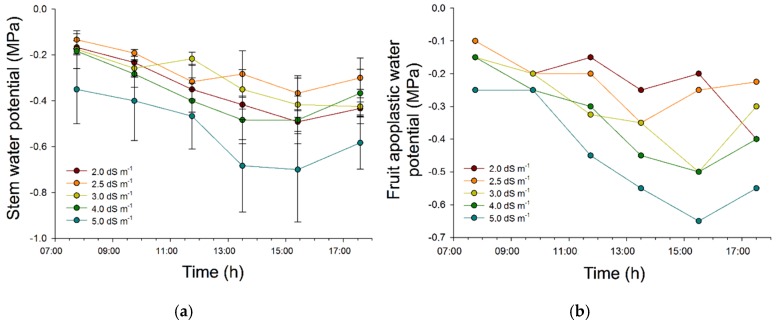
(**a**) Xylem water potential measured for stem (*n* = 3); and (**b**) apoplastic water potential measured for fruit, and this for the various salinity treatments.

As an important starting point towards metabolome data interpretation, it was verified whether the imposed salinity treatments were able to cause any physiological effects. It should be noted that although such effects may endorse the effectiveness of the imposed treatments, they do not necessarily imply significant changes of carotenoid metabolism. Based on the xylem water potential in the stem and the fruit water potential, tomato plants were found to effectively react towards imposed stress conditions through lowered water potentials (Figure 1). Significant differences (SPSS™ statistics 21.0 (IBM, Brussels, Belgium), one-way ANOVA, Dunnett’s T3, *p*-values < 0.05) for the xylem water potential were observed between the more common (2.0, 2.5 and 3.0 dS m^−1^) and extreme (4 and 5 dS m^−1^) salinity level.

In addition, the overall impact of altered salinity was verified by some secondary descriptors, *i.e.*, tomato fruit fresh weight and dry weight percentage, assessed at the picking-ready fruit stage. Average fresh weights ranged from 10.5 ± 1.0 (2.0 dS m^−1^) to 8.6 ± 0.9 (5.0 dS m^−1^) g per fruit with an almost continuous decline in average fresh weight upon increasing salinity. However, no significant differences could be revealed. Following this, the total fresh yield per plant (10 trusses) was estimated to be 980 g for the EC-treatment of 2.0 dS m^−1^, 907 g for 2.5 dS m^−1^, 875 g for 3.0 dS m^−1^, 832 g for 4.0 dS m^−^^1^, and 813 g for 5.0 dS m^−1^. These results indicate a considerable adverse impact of increased salinity on tomato fruit fresh yield. Dry matter percentages were calculated on the basis of fresh and lyophilized tomato fruit weights, and were significantly affected by altered salinity (one-way ANOVA, Tukey, *p*-values < 0.05). Higher percentages were obtained at the EC-treatments of 5.0 dS m^−1^ (11.5% ± 0.8%) and 4.0 dS m^−1^ (11.5% ± 0.7%) compared to the EC-treatments of 2.0 dS m^−1^ (10.6% ± 0.7%), 2.5 dS m^−1^ (10.4% ± 0.8%), and 3.0 dS m^−1^ (10.6% ± 0.9%). These results indicate that the more extreme salinity levels affect dry matter accumulation in tomato, as has been reported [5]. This conclusion was strengthened by the data that were obtained from the sugar and organic acid analyses (Figure S1, Supplementary Materials). Indeed, significant higher glucose and fructose concentrations (Mann–Whitney *U*, *p*-value < 0.05) were noted upon increasing salinity, and this for developmental stage S4, comparing the EC-treatment of 2.5 *versus* 4.0 and 5.0 dS m^−1^. In addition, for citric acid, concentrations levels were significantly higher (Mann–Whitney *U*, *p*-value < 0.05) for developmental stage S4 (2.5 *versus* 4.0 *versus* 5.0 dS m^−1^) and S6 (2.5 *versus* 5.0 dS m^−1^). For malic acid, significantly higher concentration levels were observed during S6, when the EC-treatment of 2.5 dS m^−1^ was compared to the treatments of 5.0 and 4.0 dS m^−1^.

### 2.2. Metabolic Fingerprinting of Tomato Fruits

Metabolic fingerprints of the developing tomato fruits comprised 21,689 monoisotopic ions (^12^C) of which the majority (69%) was acquired in positive ionization mode. The corresponding, normalized data matrix was used to screen for metabolites that are involved in the plant salinity response and carotenoid metabolism. Since these data included the ion abundances on a dry weight basis, possible concentration effects that were solely caused by osmotic stress and related impaired water uptake were excluded.

### 2.3. Metabolites Involved in the Salinity Stress Response

In order to anticipate any potential influence of fruit age on the salinity response, it was opted to generate within each individual fruit developmental stage orthogonal partial least squares to latent structures discriminant analysis (OPLS-DA) models that discriminate between salinity treatments. This strategy was justified since no significant differences (one-way ANOVA, *p*-values > 0.05) were observed in days after anthesis (DAA) between imposed salinity treatments, within each stage of development. OPLS-DA models that were compliant with the suggested validation criteria (Section 4.7) are presented in Table 1. These models were able to discriminate between two EC-treatments, whereby the treatment of 2.0 dS m^−1^ was denoted as the common reference.

**Figure 2:**
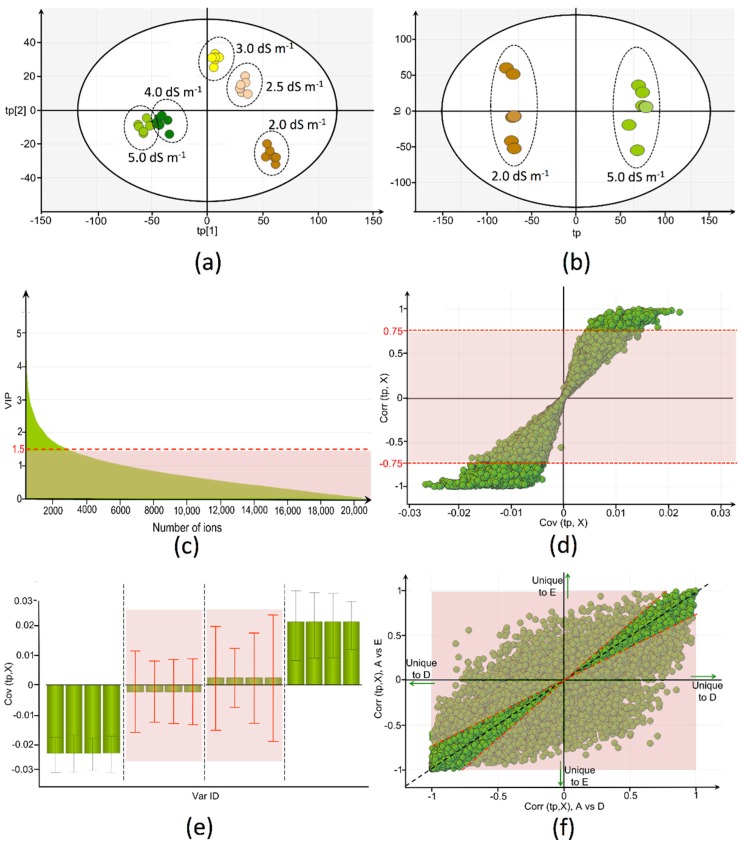
(**a**) Orthogonal partial least squares to latent structures discriminant analysis (OPLS-DA) model that was generated for the initial stage of fruit development (S1), whereby discrimination between the various electrical conductivity (EC)-treatments was achieved on the basis of the metabolic tomato fruit fingerprints. Selection of relevant ions was performed for each of the (**b**) successfully validated two-class models (e.g., 2.0 *versus* 5.0 dS m^−1^) using (**c**) variable importance in projection (VIP)-scores; (**d**) S-plot; and (**e**) loading-plot with jack-knifed confidence intervals; (**f**) the SUS-plot was used to visualize the shared information for multiple two-class OPLS-DA models. Shaded areas represent the ions that were considered irrelevant and excluded. For clarity of figures, following labeling of treatments was introduced: A, 2.0 dS m^−1^; D, 4.0 dS m^−1^; E, 5.0 dS m^−1^.

Appropriate visualization tools and associated descriptors were used for data interpretation and selection of biochemically relevant ions, respectively (Figure 2). Selection of a metabolite was based on the variable importance in projection (VIP) score (VIP > 1.5) [22], the reliability of the ion’s contribution towards the model predictive component (S-plot corr(tp, X), cutoff value of 0.75 (or −0.75)) [23], and the jack-knifed confidence interval (CIJF_JK_ (Jack-knifed confidence interval), not across zero) [24]. The above-mentioned selection criteria were applied for each OPLS-DA model. However, when multiple OPLS-DA models were generated for a particular fruit developmental stage, it was assumed that ions should only be retained if recurrent relevant along the various valid OPLS-DA models. The assumption was thus made that metabolites, which are biochemically involved in the plant salinity response, are not qualitatively differing as a function of salinity severity (on condition of effective salinity treatments). With this rationale, the shared and unique structure (SUS) plot was used to combine information from multiple models with a same reference [25]. In this study, an ion was recognized as a shared structure and retained if the corr(tp, X) ratio was between 0.75 and 1.25.

**Figure 3:**
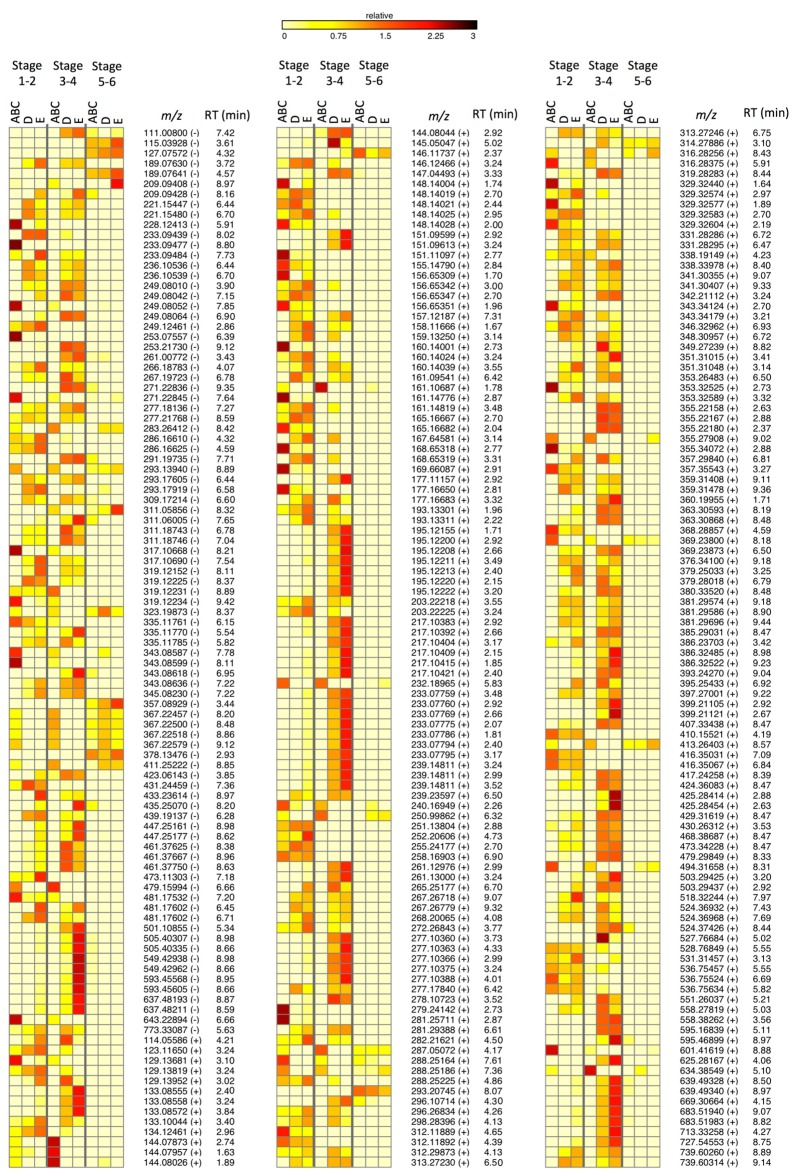
Heat map in which the relative abundances of metabolite ions, assigned a key role in the plant’s salinity response, are presented. The degree by which a metabolite’s concentration level changed, is thereby indicated by the color gradation rather than by the absolute color intensity. Each of the metabolites is characterized by a unique combination of retention time and *m/z*-value, whereby the ionization mode is indicated by the plus and minus sign. In order to simplify, it was opted to combine the results of specific EC-treatments and developmental stages.

Based on the outlined selection strategy, a large number of metabolites was retained, *i.e.*, 277 for S1, 942 for S2, 1200 for S3, 779 for S4, 742 for S5, and 84 for S6. However, evaluation of the chromatographic peak performance, especially in terms of peak shape (peak asymmetry A_S_ ≤ 1.5), was able to further reduce these numbers by excluding falsely assigned peaks (*i.e.*, artifact peaks). Eventually, 306 metabolites were assigned a key role in the tomato plant’s salinity response, *i.e.*, at the level of the fruit (Figure 3).

### 2.4. Metabolites Involved in Carotenoid Metabolism

**Figure 4:**
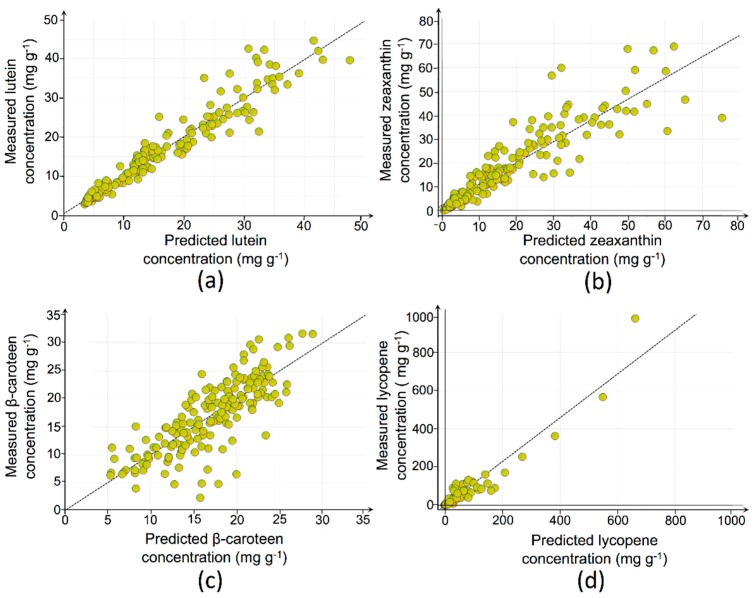
Graphical representation of the OPLS-models’ output, relating the observed and predicted *Y*-variables (*i.e.*, carotenoid concentrations). Individual valid models were generated for: (**a**) lutein; (**b**) zeaxanthin; (**c**) β-carotene; and (**d**) lycopene.

To focus on the underlying mechanisms of carotenoid metabolism and reveal involved metabolites, individual OPLS models were designed for each of the targeted carotenoids. These models intend to predict the tomato fruit carotenoid concentrations based on the inherent metabolic fingerprints (phytohormones and related metabolites). To this end, data from all fruits were employed without any restructuring or modifications. OPLS models that were used for interpretation of metabolic fingerprints and selection of relevant ions are presented in Table 2. These models were compliant with the adopted validation criteria with exception of the β-carotene model, for which *Q*^2^(*Y*) was below the set 0.5 threshold value. However, it was opted to further use this model since CV-ANOVA and permutation testing performed well. Model outputs, reflecting the predicted *versus* observed carotenoid concentration levels, are presented in Figure 4.

Selection of relevant metabolites was performed as described in the previous section (Section 2.3). Based on that selection strategy, the number of retained ions was 77 for lycopene, 163 for β-carotene, 444 for zeaxanthin, and 480 for lutein. These ions were assigned a potential involvement towards the metabolism of the respective carotenoids.

### 2.5. Potential of Altered Salinity to Improve Tomato Nutritional Quality

To determine ions that were involved in both the salinity response and carotenoid metabolism, the results from both screening approaches were matched against each other. This revealed 46 metabolites, which were ascribed an essential role in the salinity response as well as in carotenoid metabolism (Table 3). Taking into account that some metabolites are involved in the metabolism of multiple carotenoids, the following results were obtained: 34 metabolites for lutein, four for zeaxanthin, eight for β-carotene, and 32 for lycopene.

For the putative annotation of these metabolites, an integrated *in silico* based approach of chemical formula prediction by MZmine [26] and structure evaluation by MetFrag [27] was applied. The tandem mass spectrometric fragmentation (MS^2^) dataset, used for structure evaluation, was obtained by analysis of representative tomato fruit extracts (*n* = 15), thereby applying Q-Exactive^TM^ hybrid quadrupole Orbitrap-MS (Thermo Fisher Scientific, San Jose, CA, USA) [28]. Hereby, data-dependent fragmentation (dd-MS^2^) and an inclusion list were used. This list was constructed based on the theoretical masses of the metabolites of interest. Based on the identities, 10 metabolites showed a close resemblance to known phytohormones, endorsing their assigned regulating role. Hereby, especially the class of the auxins and cytokinins was strongly represented by the phytohormone-like metabolites. For both classes, various compounds have been linked to the salinity response [29] and/or carotenoid metabolism [21]. Indeed, elevated auxin concentration levels are observed upon exposing plants to salt stress, thereby delaying the deleterious effects of salts and increasing the tolerance to ions by up regulating genes that are involved in salt and osmotic alleviation [29]. Cytokinins have also been assigned a role in enhancing salt stress tolerance by a direct free radical scavenging activity and the triggering of antioxidative mechanisms [30,31]. Moreover, auxins fulfill an important (inhibiting) role in the processes of carotenoid formation and ripening [32]. For cytokinins, however, the role in carotenoid metabolism is rather limited [21].

**Figure 5:**
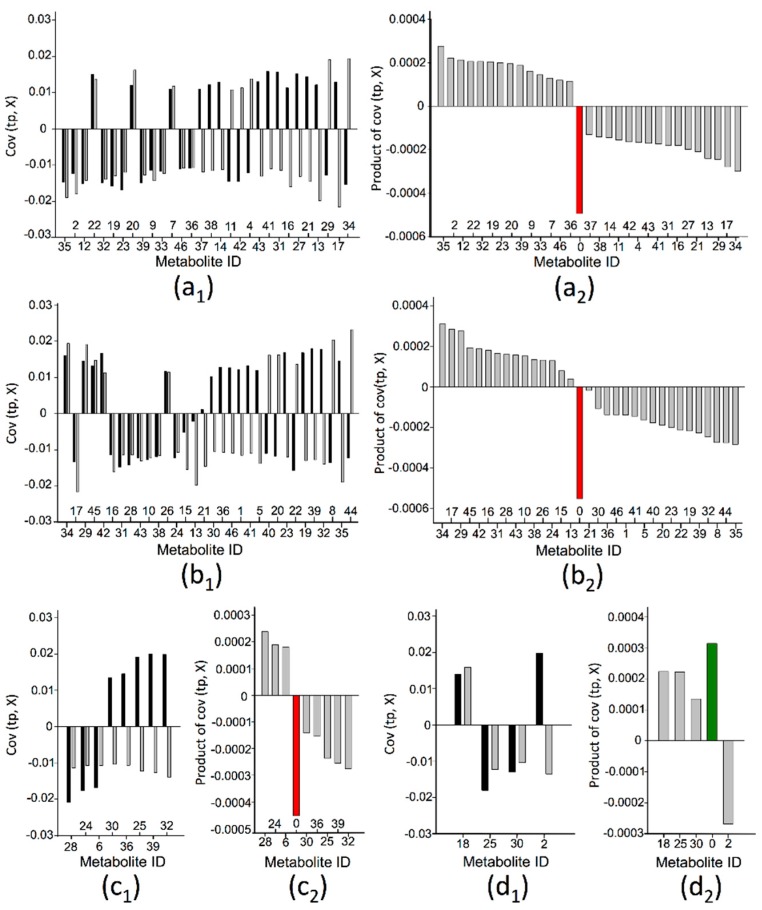
Individual covariance plots for: (**a_1_**) lutein; (**b_1_**) lycopene; (**c_1_**) β-carotene; and (**d_1_**) zeaxanthin. The covariance (effect) of a metabolite with respect to the carotenoid concentration is indicated in black. The covariance of a metabolite towards altered salinity is indicated in grey and was acquired from the OPLS-DA models, discriminating between the EC-treatments of 2.0 and 5.0 dS m^−1^. Individual plots of covariance products for: (**a_2_**) lutein; (**b_2_**) lycopene; (**c_2_**) β-carotene; and (**d_2_**) zeaxanthin. The metabolite with ID zero represents the sum of all covariance product functions. Red color indicates a total negative outcome and green color a total positive outcome.

Next, the metabolites’ changes towards altered salinity and their effects on carotenoid metabolism were integrated in order to refute or prove the potential of this agronomic strategy as a tool to promote tomato fruit nutritional quality. For this purpose, covariance plots (tp, X) were established per carotenoid (Figure 5). With respect to the salinity-related covariance, the data from the OPLS-DA models that were generated to discriminate between the EC-levels of 2.0 and 5.0 dS m^−1^ was used. As such, all developmental stages could be represented through a same model level (Table 1). In addition, if a metabolite was found significantly influenced by altered salinity in various stages of fruit development, the average covariance was used.

With respect to these covariance plots (Figure 5a_1_–d_1_), it can be stated that a metabolite with concurring covariances (*i.e.*, with identical signs) may positively contribute towards a promoted tomato fruit carotenoid content upon increased salinity. Therefore, the final effect of each metabolite was calculated as the product of both its covariances, which resulted in a new covariance function [33] (Figure 5a_2_–d_2_). Increasing the nutrient solution EC-level would thus be a useful practice if the overall positive effect outweighs the overall negative effect. Therefore, the cumulative of these new covariance functions was determined for each carotenoid (Figure 5a_2_–d_2_) and evaluated by the associated equivalent of the average metabolite effect. More specifically, the average product of covariance was determined along all metabolites and compared to this cumulative covariance. As such, a relatively small negative effect of altered salinity on the accumulation of lutein (2.6 equivalents), lycopene (3.1 equivalents), and β-carotene (2.1 equivalents) was revealed. For zeaxanthin, a relatively small positive effect (1.5 equivalents) was found.

Based on these results, it may be concluded that altered salinity has, at best, only little impact on the accumulation of carotenoids in the considered tomato cultivar. This finding was confirmed by evaluating the carotenoid concentration levels for the final stage of fruit development, for which no statistical differences (one-way ANOVA, *p*-values > 0.05) were observed between treatments (Figure S2, Supplementary Materials).

## 3. Discussion

### 3.1. Effectiveness of Imposed Salinity Treatments

This study aimed at investigating the potential of nutrient solution salinity as a strategy to promote cherry tomato carotenoid content, thereby applying an alternative and innovative research strategy. Instead of solely considering the final carotenoid concentrations, it was opted to focus on the interlinked metabolic activities that underlie carotenoid metabolism and the salt stress response. However, a true effectiveness of the imposed salinity treatments was a crucial starting-point to mark any salinity-induced changes of the metabolome. Therefore, xylem water potential was surveyed as a descriptor of water status and dehydration, proving the ability of the more extreme EC-levels (4.0 and 5.0 dS m^−1^) to induce an environmental stress condition. This finding was confirmed by the significantly higher tomato fruit dry weight percentages, which were observed under these salinities. Moreover, as fresh weights were not affected, the increased dry weight percentages are the result of activated physiological plant processes. As such, the EC-levels of 4.0 and 5.0 dS m^−1^ could be assumed to trigger tomato metabolome changes as well. These may concern signal-transduction molecules that mediate salt stress acclimation, compounds that are involved in the acclimation processes, and byproducts of stress because of a disruption of the normal homeostasis [34].

### 3.2. Impact of Salinity on the Tomato Fruit Metabolome

In the first part of this research, the overall impact of salinity on the tomato fruit metabolome was investigated, thereby taking the various stages of fruit development in consideration. After all, the metabolic composition of tomato fruit is known to strongly vary during development and ripening, which is reflected by pronounced shifts within the metabolome [35]. Therefore, any metabolic changes that are provoked by altered salinity could be expected to depend on fruit age as well. Indeed, the size of the metabolome fraction, involved in the salt stress response, was noticed to strongly vary as a function of fruit age. Although this may partially relate to fruit age and the predominant physiological processes (cell division, cell expansion, ripening, *etc.*), the number of validated OPLS-DA models (Table 1) is also important. The availability of multiple models makes it indeed possible to effectuate additional ion exclusion steps (SUS-plot), as such increasing the reliability of retained salinity-responsive ions. The varying number of OPLS-DA models along the developmental stages may relate to the climatic conditions at the time of harvest (antagonizing or enhancing the salinity effects) or varying climatic conditions at consecutive harvest moments (enlarging the variation between samples within a treatment). Eventually, the applied metabolomics strategy resulted in a joint metabolome subset of 306 metabolites, which were assigned a role in the salt stress response. Based on these metabolites and their salinity-induced intensity changes (Figure 3), two interesting findings were noted.

Firstly, the imposed salinity treatments were found to affect only a small number of metabolites during the final stages of fruit development, which is most obvious when the comparison is made with the earlier stages. This relates to the diverse processes of senescence, which strongly emerge from the moment of the breaker stage to fruit ripeness [36]. Senescence is an oxidative phenomenon, whereby the available antioxidative systems are less and less capable to neutralize all of the generated reactive oxygen species [34,37]. Within this context, environmental stress factors have been indicated to induce the production of oxygen species and inhibit the antioxidative systems, as such contributing to a more rapid senescence [38]. This may underlie the moderate impact of salinity during the final developmental stages since the adaptive responses, which antagonize the progress of senescence, have already been fully triggered and are not affected any more by salt stress. However, although this explains the small number of metabolites with pronounced intensity changes upon increased salinity (*i.e.*, with pronounced color gradients, Figure 3), it would also imply high intensities (*i.e.*, color intensities, Figure 3) for many metabolites compared to the other developmental stages. As this is not always the case, the general decrease of metabolic activity during ripening should also be regarded as a leading cause of the observed metabolite patterns [39].

A second finding relates to the metabolites’ concentration gradients, established along the various salinity treatments and evaluated within each phase of fruit development. For the early stages (S1 and S2), a substantial fraction of the salinity-responsive metabolites possesses a declining concentration profile, pointing towards a suppressive influence of salinity. This is in strong contrast with the subsequent stages (S3 and S4) for which only a minority of the metabolites shows such a gradient. Instead, most metabolites were characterized by an increasing concentration upon more intense salinities. This finding may rely on the physiological processes, underlying the cited phases of fruit development. The early stages of development are mainly driven by cell division and expansion, which are considered decisive for the final fruit size [39]. Since decreased fruit size has been reported as one of the possible effects upon salt stress [40,41], inhibition of cell division and expansion is, together with impaired water uptake, a plausible response. Therefore, it is hypothesized that some of the metabolites, adversely affected by salinity, are involved in the mitotic processes of cell division or cell wall modification during expansion [39,42]. With respect to the subsequent developmental stages, fruit maturation and ripening are the predominant processes, whereby most metabolites can be assigned an osmobalancing function [43]. Eventually, although the disruption of metabolic homeostasis is clearly indicated by Figure 3, precise details on the adjustment of metabolic pathways is not deducible. Further research, correlating direct measurements with changes in transcriptome and proteome expression is thus designated. Moreover, this would allow endorsing the presumed regulating role of the listed metabolites (Table 3), which is currently based on statistical correlations and a possible structural association with known phytohormones.

### 3.3. The Potential of Salinity to Improve Carotenoid Content

In our study, no potential of salinity to promote carotenoid accumulation in cherry tomato (cv. Juanita) was concluded. Although this concurs with the findings of other studies [16,44,45], it cannot be renounced that some studies [5,12,19,46,47] were able to clearly indicate increased tomato fruit carotenoid concentrations (up to 40%) in response to elevated salinity levels.

One of the main reasons for these conflicting results may relate to the genetic background of the assessed tomato cultivars, which holds varying salt tolerances [48]. Indeed, various key mechanisms within the plant stress response have been recognized to be universal, but their relative importance may vary from species to species, depending amongst other on the metabolic background [49]. This statement has clearly been demonstrated in the study of Caro *et al.* (1991) [50] in which normal-fruited tomato cultivars (*Solanum lycopersicum* L.) were found to be less salt tolerant compared to cherry tomatoes (*Solanum lycopersicum* L. var. cerasiforme). This would explain why in our study, which involved a cherry tomato cultivar, no effects of altered salinity on carotenoid accumulation were observed. It should hereby be noted that involvement of carotenoids in the plant’s stress response relates to the quenching of harmful singlet oxygen and peroxyl radicals that are generated in response to the stress condition [51]. However, priority might be given to other stress reducing molecular and biochemical mechanisms, including selective exclusion of ions, alteration in membrane structure, induction of antioxidative enzymes, *etc.* [52]. Taking into account these findings, a further increase of the nutrient solution EC-level (>5 dS m^−1^) would signify an appropriate measure to still achieve an enhanced carotenoid content. However, the structure of the covariance plot (Figure 5) indicates little chance on success. The near-equilibrium between the metabolites with an overall positive and negative effect would require a very unbalanced alteration of the salinity-induced metabolites following increased salinity. More specifically, a relatively high percentage of the metabolites with an overall positive effect needs to be upregulated whereas those with a negative effect need to be downregulated.

A second reason may relate to the prevailing environmental conditions during the experiment, which could interact with and influence the salinity effects [19]. It has been reported by Cuartero and Fernández-Muñoz (1999) [53] that high temperature, intense radiation, and low relative humidity enhance osmotic stress, being associated with high salt doses. In our study, no such conditions were monitored, considering the microclimate rather antagonistic towards the establishment of salt stress. However, again, based on the structure of the covariance plots, no better results are to be expected under different environmental conditions.

## 4. Materials and Methods

### 4.1. Plant Material and Growing Conditions

The experiment was carried out in a 60-m^2^ greenhouse compartment of the Institute for Agricultural and Fisheries Research (ILVO, Melle, Belgium). Cherry tomato plants (*Solanum lycopersicum* L. cv. Juanita) were obtained from Hollandplant (Bergschenhoek, the Netherlands) and introduced into the greenhouse compartment on 28 November 2013. Tomato seedlings were at that moment about four weeks old and immediately transplanted into 15-L rock wool slabs (Grodan Master, Hedehusene, Denmark) whereby a final density of about 1.9 plants m^−2^ was achieved. Temperature was regulated with heating set points of 17 °C at night and 22 °C during the day (Figure S3A, Supplementary Materials). Supplementary light was provided from 8:00 a.m. until 9:00 p.m. at a photon flux density of 50 µmol photosynthetic active radiation (PAR) m^−2^·s^−1^ when natural solar radiation reached values below 100 W·m^−2^ (Figure S3B, Supplementary Materials). Irrigation of tomato plants was realized by a drip irrigation system with one emitter per plant and comprised six irrigation sessions per day (at 6:00 a.m., 8:00 a.m., 10:00 a.m., 12:00 p.m., 2:00 p.m., and 4:00 p.m.). The duration of a session was adapted throughout the experiment in order to achieve a minimum 20% drain and prevent as such the accumulation of salts in the substrate. Side-shoots were weekly removed and trusses were pruned to 10 fruits.

### 4.2. Experimental Set-Up

**Figure 6:**
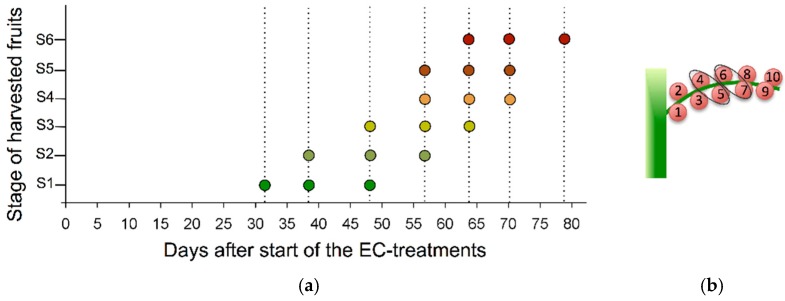
(**a**) Schematic overview of the harvest strategy, which was executed for each individual EC-treatment. Seven harvest moments, represented by the dashed lines, were considered. At each harvest moment, specific stages of fruit development were harvested, as indicated on the Y-axis and represented by the colored circles (coloring reflects developmental stage). Hereby, six stages of fruit development and ripening were defined (*i.e.*, from S1 to S6) based on days after anthesis; and (**b**) Schematic representation of the sampling and pooling strategy. Hereby the fruits that were preferred for sampling are indicated. Fruits within a same circle were pooled.

The experimental set-up included five different treatments, which concerned the salinity of the irrigated nutrient solutions. Each treatment comprised seven tomato plants (excluding border plants) whereby nutrient solution EC-levels reached values of 2.0, 2.5, 3.0, 4.0 or 5.0 dS m^−1^. In this regard, the salinity treatments of 4.0 and 5.0 dS m^−1^ were seen as the more extreme levels whereas the other treatments tend to be more common. The nutrient solution with an EC-level of 2.5 dS m^−1^ consisted of (in mM, or in µM in case of *) 13.6 NO_3_^−^, 4.7 Ca^2+^, 3.6 K^+^, 3.5 SO_4_^2−^, 2.3 Mg^2+^, 1.2 H_2_PO_4_^−^, 1.0 NH_4_^+^, 0.2 Fe^2+^, 15.9* Na^+^, 10.1* Mn^2+^, 7.5* B^3+^, 5.0* Zn^2+^, 0.8* Cu^2+^, and 0.5* Mo^2+^, which is common practice in commercial tomato production. This standard nutrient solution was used to prepare the solutions with variant EC-levels by either dilution with rainwater or addition of appropriate amounts of NaCl/CaCl_2_ (2:1, molar basis) [54]. Initially, all tomato plants were irrigated with the standard nutrient solution for five days after which the intended EC-levels were gradually established over a period of 10 days. As such, final EC-levels were reached at 12 December 2013 and maintained till the end of the experiment, *i.e.*, 4 March 2014. Harvest of the fruits was performed as illustrated in Figure 6a and started on 7 January 2014. Six different stages of fruit development and ripening were considered and theoretically defined according to the DAA. More specifically, the desired number of DAA for stage 1 (S1), stage 2 (S2), stage 3 (S3), stage 4 (S4), stage 5 (S5) and stage 6 (S6) was respectively set at 15, 25, 35, 45, 55 and 65. It was opted for this objective measure as a color-based harvest strategy could be biased through the effects of imposed salinity treatments. For each stage, 12 tomato fruits were collected per treatment whereby the fruits originated from three different trusses (four fruits per truss), coming from three different plants. Harvest of fruits, which were set first or last within a truss, was avoided. The four fruits from each single truss were pooled per two (based on similar DAA), which thus resulted in two sampling entities per truss (Figure 6b). Harvest of fruits during the various harvest moments was always carried out at a similar time point (approximately at 5:00 p.m.). The consecutive cutting, lyophilization, grinding and sieving of each entity resulted in a homogenous powder, which allowed representative sampling. Plant material was then kept cold (−20 °C) and shielded from light until analysis, in order to prevent degradation of any fruit compound.

### 4.3. Chemicals and Reagents

The carotenoid analytical standard all*-trans*-β-carotene was from Sigma-Aldrich Co. (St. Louis, MO, USA), all*-trans*-lycopene from Phytolab GmbH & Co. KG (Vestenbergsgreuth, Germany), all-*trans*-lutein from Extrasynthese (Genay, France), and all*-trans*-zeaxanthin from TRC Inc. (Eching, Germany). The internal standard β-apo-8’-carotenal was obtained from Sigma-Aldrich Co. All phytohormone analytical and deuterium-labeled internal standards were obtained from OlchemIm Ltd. (Olomouc, Czech Republic). Reagents were of analytical grade when used for extraction purposes and of liquid chromatography – mass spectrometry grade for (ultra) high performance liquid chromatography (UHPLC)-Orbitrap-MS applications. They were purchased from VWR International and Thermo Fisher Scientific Inc. (San Jose, CA, USA), respectively. Ammonium acetate and formic acid were obtained from VWR International and magnesium carbonate was from Sigma-Aldrich Co. Ultrapure water was supplied by usage of a purified-water system (VWR International).

### 4.4. Targeted Analysis of Four Tomato Fruit Carotenoids

Extraction and targeted high-performance liquid chromatography Exactive™ Orbitrap-MS analysis of tomato fruit carotenoids was performed according to Van Meulebroek *et al.* (2014) [55] and was used to determine the concentration levels of zeaxanthin, β-carotene, lycopene, and lutein. Carotenoid concentrations were thereby calculated using matrix-matched calibration curves whereby an internal standard (β-apo-8’-carotenal) was used to counteract potential fluctuations during analysis.

### 4.5. Untargeted Analysis of Phytohormones and Related Metabolites

Extraction and detection of tomato fruit phytohormones and related metabolites were performed as described by van Meulebroek *et al.* (2012) [56]. The untargeted analysis was founded on the generic nature of the applied extraction procedure and the metabolic screening possibilities of the employed single-stage Exactive™ Orbitrap mass spectrometer (Thermo Fisher Scientific, San Jose, CA, USA). System stability during mass spectrometric analysis was verified using three calibration curves [56], which were run at different time-points during analysis of samples. These curves included six concentration levels and were established for eight phytohormones. For each of these phytohormones, coefficients of variance were calculated per concentration level and then averaged. For this purpose, area ratios were considered whereby either d_6_-abscisic acid or d_7_-N^6^-benzyladenine was used as internal standard. This led to the following results: 10.8% for N^6^-benzyladenine, 8.9% for gibberellic acid, 7.9% for (±)-*cis*, *trans* abscisic acid, 7.6% for indol-3-acetic acid, 6.1% for epibrassinolide, 5.3% for salicylic acid, 4.74% for *trans*-zeatin, and 2.1% for jasmonic acid. As these values were below 15%, appropriate stability during analysis was concluded [23].

### 4.6. Establishment of Tomato Fruit Metabolic Fingerprints

The acquired full-scan data from the untargeted analyses were processed using Sieve™ (Thermo Fisher Scientific Inc., San Jose, CA, USA) whereby non-differential single class analysis was applied. This strategy aimed at defining the metabolites in terms of *m*/*z*-value, retention time, and signal abundance. The main parameter settings included a scan range of 100–800 Da, an *m*/*z*-width of 10 ppm, a maximum peak width of 0.5 min, a minimum intensity threshold of 50,000 arbitrary units, and a maximum of 30,000 frames (*i.e.*, unique combinations of retention time and *m*/*z*-value). The generated data matrix was normalized by the signal intensities of two deuterium-labeled internal standards [25], *i.e.*, d_6_-abscisic acid (for negative ions) and d_7_-N^6^-benzyladenine (for positive ions), both supplemented prior to extraction.

### 4.7. Screening for Metabolites Involved in the Salinity Stress Response

Multivariate data analysis was performed by SIMCA™ 13 software (Umetrics, Malmö, Sweden) and aimed at revealing those metabolites that are involved in the plant salinity stress response. For this purpose, data were reorganized into six data clusters, whereby each individual cluster contained the data of a well-defined stage of fruit development (*i.e.*, S1, S2, S3, S4, S5, and S6). Within each cluster and thus for each stage of fruit development, potential effects from the EC-treatments on the metabolic fingerprints were investigated. OPLS-DA [57] was applied to model these multiple classes, *i.e.*, to identify a relationship between the LC-MS data (*X*-variables) and the EC-treatments, which were described as discrete values (*Y*-variables). Prior to OPLS-DA modeling, pareto-scaling (1/√SD, where SD is the standard deviation) and automatic transformation of the data were performed to standardize the range of independent *X*-variables and induce normality, respectively [25]. Validity of the models was verified by CV-ANOVA (*p*-values < 0.05), permutation testing (*n* = 100), and three model characteristics (*i.e.*, *R*^2^(*X*), *R*^2^(*Y*) and *Q*^2^(*Y*), calculated by 7-fold cross-validation) [58,59,60].

### 4.8. Screening for Metabolites Involved in Carotenoid Metabolism

Deepening the knowledge about carotenoid metabolism was founded on modeling the interrelationship between the tomato fruit fingerprints and their inherent carotenoid concentrations. Multivariate data analysis was performed by SIMCA™ 13 software whereby OPLS models were specifically constructed for each of the targeted carotenoids. These models intend to explain and predict one quantitative *Y*-variable (carotenoid concentration) from the *X*-matrix (tomato metabolic fingerprints). A same practice with respect to data preprocessing (scaling and transformation) and model validation as earlier described was applied.

### 4.9. Effectiveness of Imposed Salinity Treatments

To verify any actual effects of the applied salt treatments, stem xylem water and fruit total water potential were determined with a pressure chamber (PMS Instruments Co., Corvallis, OR, USA) on 12 March 2014. Xylem water potential of the stem was measured on leaves that were enclosed in aluminum envelopes for one hour prior to measuring their water potential.

## 5. Conclusions

Within this study, a metabolic fingerprinting strategy was applied to define the overall impact of salinity on the tomato fruit metabolome and assess its implications on carotenoid accumulation. Hereby, a substantial fraction of the fruit metabolome was found affected by altered salinity, with marked differences depending on the fruit’s developmental stage and the underlying physiological processes. Insights on these interrelations may enclose interesting opportunities to improve tomato production and overall quality, including nutritional value. In this study, the selected fingerprinting strategy was applied to determine the true potential of altered salinity in enhancing carotenoid accumulation, thereby moving away from typical black box scenarios. Although no or only little potential was concluded for the concerned cherry tomato cultivar, the proposed strategy has the ability to both elucidate and exploit the potential of agronomic measures in promoting nutritional quality. Indeed, the dynamic behavior of involved metabolites towards changing growth conditions allows a more rapid unraveling of the interactive effects between genetic background, agronomic settings, and environment compared to carotenoids. Measured carotenoid concentration levels can indeed be regarded as the cumulative outcome of all possible (interactive) effects that occurred during the passed phases of fruit development and ripening. This novel approach may thus enclose significant value in modeling tomato fruit nutritional quality and provides interesting insights on the metabolic salinity responses during tomato fruit development and ripening. However, further functional research is designated, correlating direct measurements with changes in transcriptome and proteome expression.

## Figures and Tables

**Table 1:** Valid orthogonal partial least squares to latent structures discriminant analysis (OPLS-DA) models that were able to discriminate between electrical conductivity (EC)-treatments. Validation of the models was performed by CV-ANOVA (*p*-value < 0.05), permutation testing and three model characteristics ((*R*^2^(*X*), *R*^2^(*Y*), and *Q*^2^(*Y*)) (SIMCA 13, Malmö, Sweden). All of the presented models performed well for CV-ANOVA and permutation testing. The following labeling of EC-treatments was introduced: A, 2.0 dS m^−1^; B, 2.5 dS m^−1^; C, 3.0 dS m^−1^; D, 4.0 dS m^−1^; E, 5.0 dS m^−1^.

Model Description		*R*^2^(*X*)	*R*^2^(*Y*)	*Q*^2^(*Y*)
S1	A *versus* C	0.852	1.000	0.824
	A *versus* D	0.419	0.990	0.900
	A *versus* E	0.694	0.996	0.952
S2	A *versus* D	0.785	1.000	0.940
	A *versus* E	0.658	0.998	0.953
S3	A *versus* E	0.700	0.999	0.890
S4	A *versus* D	0.721	0.999	0.965
	A *versus* E	0.696	1.000	0.891
S5	A *versus* D	0.551	0.996	0.865
	A *versus* E	0.562	0.999	0.861
S6	A *versus* C	0.626	0.992	0.884
	A *versus* D	0.528	0.999	0.935
	A *versus* E	0.585	0.938	0.938

**Table 2:** OPLS models that were able to predict tomato fruit carotenoid concentrations based on metabolic fingerprints. Validation of these models was performed based on CV-ANOVA (*p*-value < 0.05), permutation testing and three model characteristics ((*R*^2^(*X*), *R*^2^(*Y*), and *Q*^2^(*Y*)). All of the presented models performed well for CV-ANOVA and permutation testing.

Model Description	*R*^2^(*X*)	*R*^2^(*Y*)	*Q*^2^(*Y*)
Lutein	0.564	0.957	0.915
Zeaxanthin	0.660	0.906	0.698
β-Carotene	0.592	0.647	0.308
Lycopene	0.672	0.988	0.929

**Table 3:** Metabolites that were assigned a role in both carotenoid metabolism and the salinity response. These metabolite ions were labeled with an identification number (*i.e.*, Metabolite ID) for further graphical representations and putatively annotated, thereby assigning a best identity match (PubChem database compound identifier CID). The fragmentation ratio was calculated as the ratio of the number of matching fragments of the CID-compound *versus* the highest number of matching fragments among all candidate structures. *^a^* Data-dependent tandem mass spectrometry (dd-MS^2^) analysis using an inclusion list was performed as soon as the multivariate data analysis was completed and essential information about the relevant metabolites was gathered. However, during this time span, some metabolites were degraded. As such, identification efforts were limited to determining the elemental composition on the basis of accurate mass and the ^13^C isotope profiles.

Metabolite ID	Ionization Mode	*m/z*-Value	Retention Time (min)	CID	Fragmentation Ratio (%)	Elemental Composition
1	+	476.2991	7.28	11698581	100.0	C_27_H_41_N_1_O_6_
2	+	269.2096	7.13	21720337	85.0	C_16_H_28_O_3_
3	+	409.1486	5.39	44144278	81.3	C_20_H_24_O_9_
4	+	711.2440	4.63	10349855	100.0	C_33_H_42_O_17_
5	+	576.2689	3.89	46242589	64.2	C_25_H_42_N_3_O_10_P
6 *^a^*	+	785.8416	4.73	n.a.		
7 *^a^*	+	572.4492	8.36	n.a.		
8	+	311.2166	6.35	20686694	57.1	C_13_H_30_N_2_O_6_
9	+	337.2015	2.99	19511475	86.7	C_20_H_24_N_4_O
10 *^a^*	+	602.4972	6.26	n.a.		
11	+	355.1776	4.61	15690336	100.0	C_15_H_30_O_7_S
12	+	275.0906	4.70	28848874	41.7	C_13_H_12_N_3_O_4_
13	+	596.4120	4.63	44409845	70.0	C_33_H_57_NO_8_
				10416091		
14	+	531.2604	3.49	3188562	50.0	C_30_H_34_N_4_O_5_
				3188563		
15	+	528.3285	5.94	44275272	50.0	C_24_H_50_NO_9_P
16	+	530.2776	6.70	24999541	50.0	C_30_H_35_N_5_O_4_
17	+	485.1479	5.26	314405	100.0	C_22_H_28_O_10_S
18	+	221.1138	5.98	18519108	100.0	C_8_H_16_N_2_O_5_
19	+	348.2155	6.20	22997503	76.4	C_20_H_29_NO_4_
20	+	390.1299	4.58	46657456	78.6	C_19_H_15_N_7_O_3_
21	+	462.1048	2.85	1190411	100.0	C_14_H_19_N_7_O_9_S
22	+	410.1552	4.19	27682727	71.4	C_19_H_27_N_3_O_3_S_2_
				32647280		
23	+	377.1055	3.60	45420283	70.5	C_19_H_20_O_6_S
24	+	775.3393	5.67	21630904	66.7	C_36_H_54_O_18_
25	+	188.0636	4.14	89915	73.7	C_11_H_9_NO_2_
26	−	163.0394	3.26	691	100.0	C_9_H_8_O_3_
27 *^a^*	−	565.0488	5.52	n.a.		
28	−	773.3309	5.64	11767898	76.9	C_43_H_46_N_6_O_8_
29	−	736.3310	3.86	21681260	50.0	C_40_H_51_NO_2_
30 *^a^*	−	115.0393	3.61	n.a.		
31	−	431.2446	7.37	12133739	85.8	C_25_H_36_O_6_
32	−	378.1348	2.94	25232866	83.2	C_22_H_21_NO_5_
33	−	425.1469	5.38	36942227	75.0	C_21_H_22_N_4_O_6_
34	−	569.0984	6.55	23406912	77.8	C_26_H_26_N_4_O_5_S_3_
35	−	603.2794	8.08	44282378	100	C_32_H_44_O_11_
36	−	189.0764	4.58	26339616	85.6	C_8_H_14_O_5_
37	−	249.1246	2.86	45809090	100.0	C_13_H_18_N_2_O_3_
				18108416		
				43403806		
				43421263		
				43392898		
38	−	286.1661	4.33	21403706	85.7	C_14_H_25_NO_5_
39	−	378.1351	2.65	45898135	93.3	C_22_H_21_NO_5_
40	−	461.0163	4.36	15614249	87.5	C_12_H_21_NO_4_
41	−	242.1400	5.52	30309738	36.4	C_13_H_17_N_5_
42	−	361.0696	3.29	1014968	100.0	C_17_H_18_N_2_O_3_S_2_
43	−	463.2711	7.37	10457172	66.7	C_26_H_40_O_7_
				9825709		
				9847284		
44	−	486.0865	5.67	30663446	71.4	C_24_H_17_N_5_O_5_S
45	−	777.2091	5.12	20488220	100.0	C_35_H_36_N_7_O_12_S
46	−	189.0764	4.83	23399204	71.4	C_8_H_14_O_5_

n.a.: not available.

## Supplementary Materials

Supplementary materials can be found at http://www.mdpi.com/1422-0067/17/6/821/s1.

## Abbreviations

The following abbreviations are used in this manuscript:

EC	Electrical conductivity
MS	Mass spectrometry
OPLS	Orthogonal partial least squares to latent structures
DA	Discriminant analysis
DAA	Days after anthesis
VIP	Variable importance in projection
CIJF_jk_	Jack-knifed confidence interval
SUS	Shared and unique structure
dd-MS^2^	data-dependent fragmentation
CID	Compound identifier
