# Supplementary material for: Metabolic Fingerprinting to Assess the Impact of Salinity on Carotenoid Content in Developing Tomato Fruits
Source: Int J Mol Sci. 2016 May 26;17(6):821. doi: 10.3390/ijms17060821 (PMC4926355; doi:10.3390/ijms17060821)
Supplement: Supplementary file 1 [file ijms-17-00821-s001.pdf]

Int. J. Mol. Sci. **2016**, *17*, 821; doi:10.3390/ijms17060821

Supplementary Materials: Metabolic Fingerprinting to Assess the Impact of Salinity on Carotenoid Content in Developing Tomato Fruits

Lieven Van Meulebroek, Jochen Hanssens, Kathy Steppe and Lynn Vanhaecke

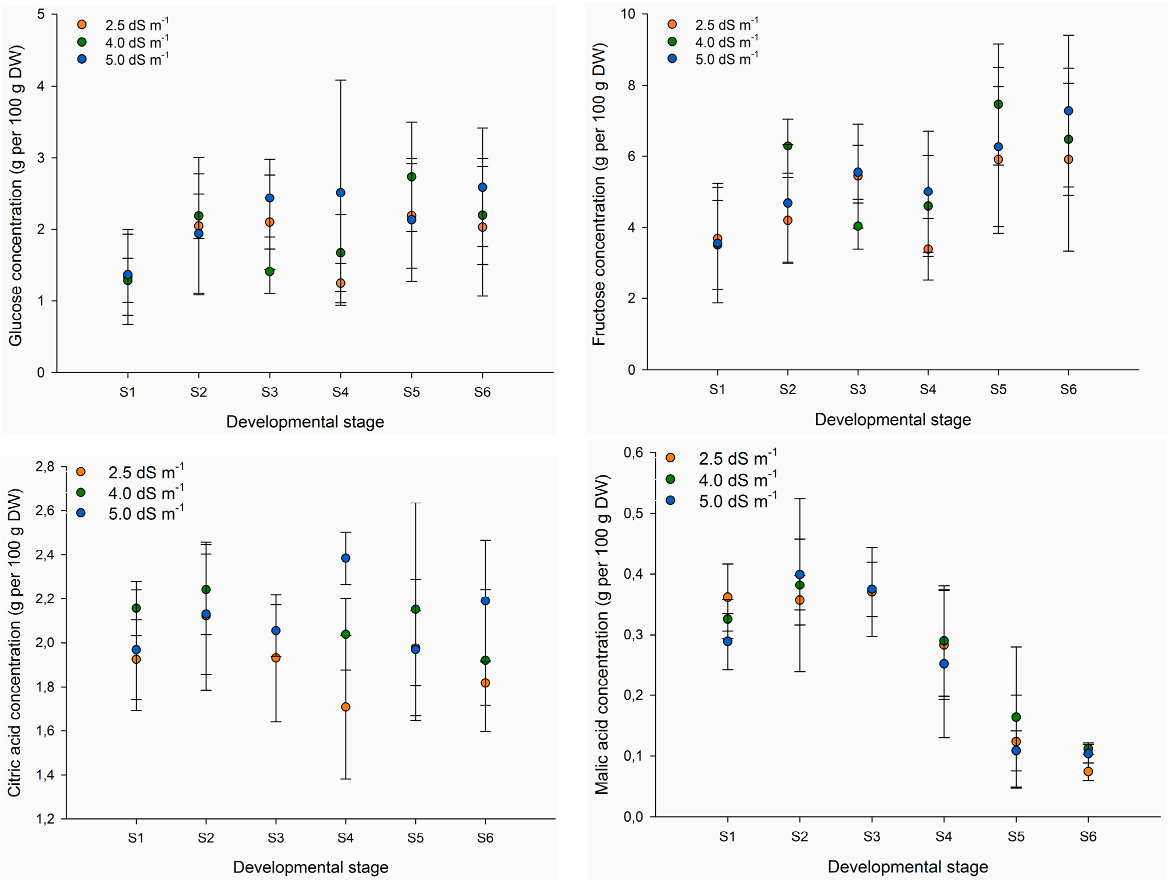

Figure S1. Measured average concentrations (g per 100 g dry weight (DW)) for glucose, fructose, citric acid, and malic acid during fruit development (developmental stages S1–S6) and under different (electrical conductivity) EC-treatments (2.5, 4.0 and 5.0 decisiemens (dS) m^{-1}). Each measurement point represents the average of 6 samples, with each sample being the homogenized sample of two fruits. Measurement variation is indicated by the standard deviation.

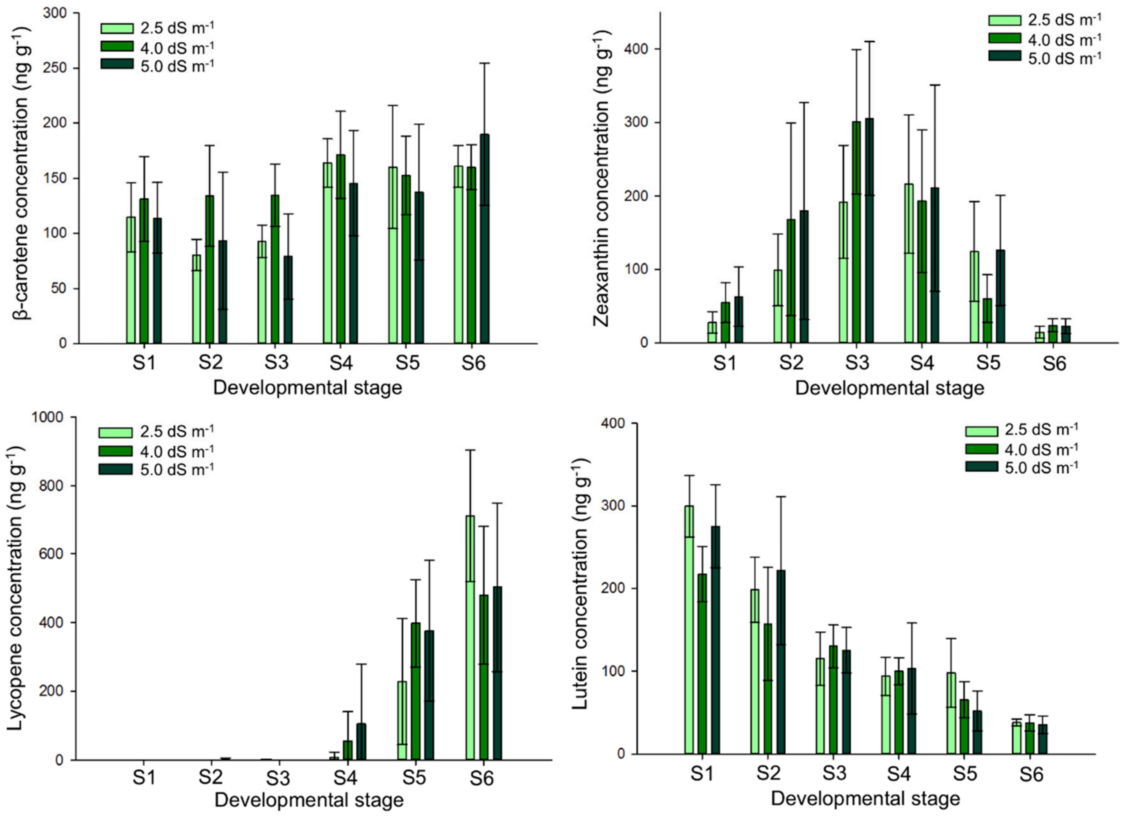

Figure S2. Carotenoid concentration levels (on a dry weight basis) for the various developmental stages and EC-treatments of 2.5, 4.0 and 5.0 dS m^{-1} (n = 6, standard deviation).

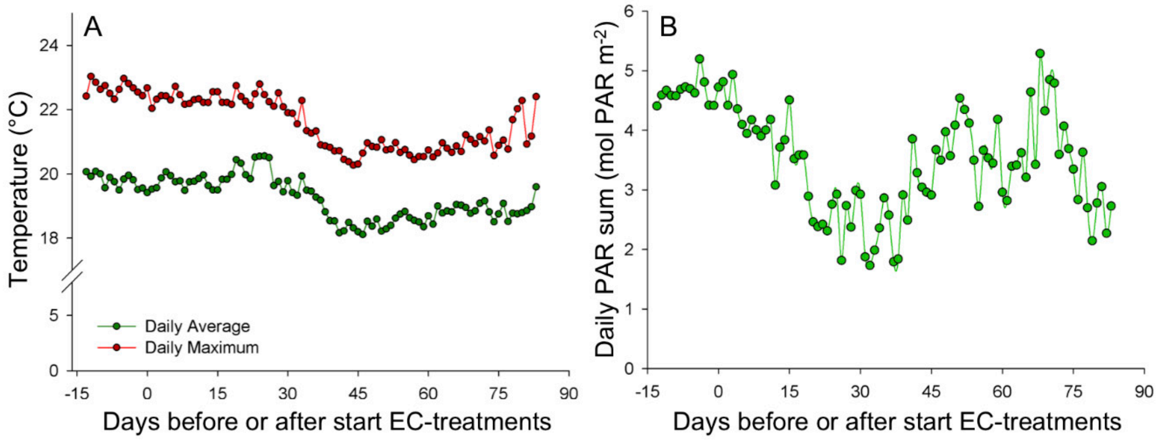

Figure S3. Average and maximum daily air temperatures (**A**), recorded inside the greenhouse compartment during the experiment; the daily photosynthetic active radiation (PAR) sum (**B**) was determined as well and comprised natural and artificially supplemented PAR-radiation. The microclimatic data are expressed in function of the number of days after or before the actual start of the EC-treatments, *i.e.*, when nutrient solutions reached their final EC-levels (12 December 2013).
